# Afucosylated anti-canine CD20 antibody combined with cyclophosphamide, doxorubicin, vincristine, and prednisone chemotherapy in dogs with B-cell lymphoma

**DOI:** 10.1093/jvimsj/aalaf039

**Published:** 2026-01-21

**Authors:** Takuya Mizuno, Kei Harada, Masanao Ichimata, Ryuzo Katayama, Yukinari Kato, Toshinori Shiga, Toshihiro Tsukui, Hiroto Toyoda, Eri Fukazawa, Fukiko Matsuyama, Masaya Igase, Tetsuya Kobayashi

**Affiliations:** Laboratory of Molecular Diagnostics and Therapeutics, Joint Faculty of Veterinary Medicine, Yamaguchi University, Yamaguchi 753-8515, Japan; Research Institute for Cell Design Medical Science, Yamaguchi University, Yamaguchi 753-8515, Japan; Japan Small Animal Cancer Center, Affiliated with the Japan Small Animal Medical Center Foundation, Tokorozawa, Saitama 359-0023, Japan; Japan Small Animal Cancer Center, Affiliated with the Japan Small Animal Medical Center Foundation, Tokorozawa, Saitama 359-0023, Japan; Japan Small Animal Cancer Center, Affiliated with the Japan Small Animal Medical Center Foundation, Tokorozawa, Saitama 359-0023, Japan; Japan Small Animal Cancer Center, Affiliated with the Japan Small Animal Medical Center Foundation, Tokorozawa, Saitama 359-0023, Japan; Department of Antibody Drug Development, Tohoku University Graduate School of Medicine, Miyagi 980-8575, Japan; Zenoaq Inc., Fukushima 963-0102, Japan; Zenoaq Inc., Fukushima 963-0102, Japan; Japan Small Animal Cancer Center, Affiliated with the Japan Small Animal Medical Center Foundation, Tokorozawa, Saitama 359-0023, Japan; Japan Small Animal Cancer Center, Affiliated with the Japan Small Animal Medical Center Foundation, Tokorozawa, Saitama 359-0023, Japan; Japan Small Animal Cancer Center, Affiliated with the Japan Small Animal Medical Center Foundation, Tokorozawa, Saitama 359-0023, Japan; Laboratory of Molecular Diagnostics and Therapeutics, Joint Faculty of Veterinary Medicine, Yamaguchi University, Yamaguchi 753-8515, Japan; Research Institute for Cell Design Medical Science, Yamaguchi University, Yamaguchi 753-8515, Japan; Japan Small Animal Cancer Center, Affiliated with the Japan Small Animal Medical Center Foundation, Tokorozawa, Saitama 359-0023, Japan; Japan Small Animal Cancer Center, Affiliated with the Japan Small Animal Medical Center Foundation, Tokorozawa, Saitama 359-0023, Japan

**Keywords:** antibody therapy, B-cell depletion, cancer, chimeric antibody, dogs

## Abstract

**Background:**

B-cell lymphoma in dogs is a common hematopoietic malignancy, often treated with cyclophosphamide, doxorubicin, vincristine, and prednisone (CHOP)-based chemotherapy, but long-term outcomes remain suboptimal. Although CD20 targeting has improved outcomes in humans with non-Hodgkin’s lymphoma, it remains challenging in dogs because of the lack of effective anti-CD20 antibodies.

**Hypothesis/Objectives:**

We aimed to assess the safety, efficacy, and B-cell depletion kinetics of a novel afucosylated chimeric anti-canine anti-CD20 antibody (4E1-7-B_f) combined with CHOP chemotherapy in dogs with untreated B-cell lymphoma.

**Animals:**

Thirteen client-owned dogs with high-grade B-cell lymphoma.

**Methods:**

In this open-label, single-arm, single-center clinical trial, dogs received 4E1-7-B_f with CHOP chemotherapy. Treatment response was assessed using the Veterinary Cooperative Oncology Group criteria, whereas progression-free survival (PFS), overall survival (OS), and adverse events (AEs), and peripheral B-cell kinetics were evaluated.

**Results:**

All 13 dogs achieved complete response (CR), with a median time to CR of 3 weeks. The median PFS and OS were 340 (95% confidence interval [CI], 87-417) and 458 (95% CI, 196–not estimable) days, respectively. The 1- and 2-year survival rates were 69.2% and 38.9%, respectively. Most AEs were mild to moderate. B-cell depletion lasted for > 200 days in most dogs, with some remaining B-cells deficient for over 300 days.

**Conclusions and clinical importance:**

The combination of 4E1-7-B_f with CHOP chemotherapy showed promising efficacy and prolonged B-cell depletion. Although direct comparisons cannot be made because of the single-arm design, the results suggest a potential benefit over historical CHOP data. Additional randomized controlled trials are needed to confirm these findings.

## Introduction

B-cell lymphoma is one of the most common hematopoietic malignancies in dogs, accounting for 60%-80% of all lymphoma cases in dogs. ^1^ This disease bears similarities with non-Hodgkin’s lymphoma in humans, making it a valuable model for comparative oncology studies.^1^ B-cell lymphomas in dogs typically are treated with cyclophosphamide, doxorubicin, vincristine, and prednisone (CHOP)-based multiagent chemotherapy. Although this approach has been the standard for decades, its therapeutic outcomes are suboptimal, with a median survival time of approximately 1 year. The 2-year survival rate is approximately 50% in dogs achieving complete remission, but only 20% overall.^3^ After relapse, various salvage protocols are used, but response rates remain unsatisfactory.^4^^,^^5^ In recent years, novel agents such as rabacfosadine (Tanovea-CA1) and verdinexor (Laverdia-CA1) have been approved for treatment, but they have not substantially improved long-term survival or resulted in durable remissions.^5^^,^^6^ A recent pilot study demonstrating the feasibility and safety of allogeneic hematopoietic stem cell transplantation in dogs with high-grade B-cell lymphoma reported a long-term disease-free survival of > 4 years in 89% of dogs treated in first remission.^7^ Therefore, innovative therapeutic strategies beyond conventional cytotoxic agents (eg, adoptive cell-based therapy, marrow or stem cell transplants, monoclonal antibody treatments) are urgently needed.

Targeting CD20, an antigen widely found during B-cell differentiation and an expressed in B-cell neoplasms, has been a cornerstone of antibody-based therapy in human medicine. Rituximab, the first anti-CD20 monoclonal antibody, was approved for the treatment of diffuse large B-cell lymphoma (DLBCL) in combination with rituximab and CHOP (R-CHOP) therapy, significantly improving patient outcomes.^8^ Since its approval, second- and third-generation CD20-targeting antibodies have been developed, designed to enhance antibody-dependent cellular cytotoxicity (ADCC), complement-dependent cytotoxicity (CDC), or pharmacokinetic properties.^9^ These antibodies have been administered in patients with hematologic malignancies and autoimmune diseases, such as rheumatoid arthritis and multiple sclerosis.^10^^,^^11^ However, the therapeutic potential of CD20-targeting antibodies in veterinary medicine has remained limited because of species-specific differences in CD20 antigenicity. For example, rituximab does not cross-react with canine CD20,^12^ indicating the need for a species-specific antibody. Efforts to produce anti-canine CD20 antibodies have been reported,^13–15^ but none are commercially available or widely used. Recently, 1E4-cIgGB, a novel anti-canine CD20 antibody, demonstrated promising preclinical activity. Its clinical efficacy however remains difficult to interpret because it was combined with investigational agents such as KPT-9274 and RV1001, which are not standard treatments for lymphoma in dogs, unlike the CHOP regimen used in our study.^16^

In our previous study, we developed 4E1-7-B_f, an afucosylated chimeric anti-canine CD20 antibody that showed potent strong ADCC and weak CDC activities.^17^ In preclinical studies, a single IV dose of 5 mg/kg completely depleted peripheral blood B-cells within 24 h, with the effect persisting for 2-3 weeks. Building on these findings, we aimed to evaluate the safety and therapeutic efficacy of 4E1-7-B_f in combination with CHOP therapy for B-cell lymphomas in dogs to integrate targeted immunotherapy into current treatment paradigms and address a critical unmet need in veterinary oncology.

## Materials and methods

### Study design

A prospective, single-arm, open-label, nonrandomized, single-center clinical trial was conducted in dogs with previously untreated high-grade B-cell lymphomas. During the initial screening, each dog underwent a physical examination, baseline CBC, serum biochemistry panel, urinalysis, and lymph node aspiration cytology. For diagnostic confirmation, cytology was performed by a single pathologist, except in 2 cases where an additional pathological diagnostic analysis was performed alongside aspiration cytology. Lymph node aspiration samples were further analyzed by flow cytometry to assess CD3, CD21, and CD20 expression. The size of lymphoma cells was evaluated using forward scatter in flow cytometry and classified as intermediate to large by comparison with mature normal lymphocytes. Dogs were eligible if they were diagnosed with CD21+ and CD20+ large-to-intermediate B-cell lymphoma based on flow cytometry and cytological evaluation of lymph node aspirates. Enrollment criteria included peripheral lymphadenopathy and adequate organ function, confirmed by standard laboratory tests (CBC, serum biochemistry, and urinalysis). However, specific numerical thresholds for organ function tests were not strictly defined, because some laboratory abnormalities (eg, mild increases in liver enzyme activities or changes in white blood cell counts) may result from lymphoma itself rather than organ dysfunction. Therefore, decisions regarding eligibility were made at the discretion of the attending clinician, based on the overall clinical picture and the judgment that organ function was sufficient to safely initiate treatment. Dogs were enrolled between January 2020 and December 2023 and met the inclusion criteria. All participating dog owners provided written informed consent before enrollment.

Tumor staging was performed according to the World Health Organization clinical staging system for lymphoma in domestic animals.^4^ In our study, fine-needle aspiration (FNA) of the spleen, liver, or both was performed when abnormalities were detected on abdominal ultrasonography. Cases with cytologically confirmed infiltration of either the liver or spleen were classified as stage IV. In contrast, bone marrow aspiration was not routinely performed in all cases. Therefore, stage V designation was based on the identification of abnormal lymphocytes in the peripheral blood smear, as assessed by a board-certified clinical pathologist. Specifically, dogs were classified as stage V when intermediate-to-large lymphoid cells with atypical morphology were observed in the blood smear, consistent with neoplastic infiltration, regardless of whether bone marrow evaluation was performed. This approach reflects the practical limitations and ethical considerations of invasive sampling in client-owned dogs, while still aligning with established diagnostic criteria for lymphoma staging.

The exclusion criteria were as follows: pregnancy or lactation, severe health conditions (general condition score^18^ ≥3), life expectancy <6 weeks, and any current medications that could interfere with the study’s toxicity or antitumor efficacy assessments, such as immunosuppressive doses of corticosteroids and nonsteroidal anti-inflammatory drugs. The study protocol was approved by the Japan Small Animal Medical Center Ethical Committee.

### Treatment protocol

All enrolled dogs received anti-CD20 antibody in combination with the modified University of Wisconsin 25-week (UW25)-based CHOP chemotherapy regimen (Table 1). The anti-CD20 agent was an afucosylated canine chimeric antibody (4E1-7-B_f) formulated as a 5 mg/mL solution and stored at –20°C.^17^ The CHOP therapy followed the modified UW25 protocol, with vincristine, cyclophosphamide, doxorubicin, and prednisolone at doses of 0.7, 250, 30 (1.0 mg/kg for dogs weighing < 15 kg), and 0.5-1.0 mg/kg (discontinued within the first 4 weeks), respectively. 4E1-7-B_f was administered on the same day as vincristine, except in week 1. Before 4E1-7-B_f administration, each dog received 1 mg/kg diphenhydramine and 1 mg/kg prednisolone sodium succinate IV to mitigate infusion reaction. The antibody was diluted in 0.9% NaCl to a concentration of 1 mg/mL and infused over 2-3 h with gradually increasing rate. The infusion was initiated at 1 mg/kg/h and increased by 1 mg/kg/h every 30 min until a maximum rate of 5 mg/kg/h was reached.

**Table 1:** CD20–CHOP protocol dosing schedule.

	Week															
	1	2	3	4	6	7	8	9	11	13	15	17	19	21	23	25
**Vincristine 0.7 mg/m^2^, IV**	●		●		●		●		●		●		●		●	
**Cyclophosphamide 250 mg/m^2^, IV**		●				●				●				●		●
**Doxorubicin 30 mg/m^2^^a^, IV**				●				●				●				
**Prednisone, PO**	●	●	●	●												
**4E1-7-B_f, 5 mg/kg, IV**			●		●		●		●		●		●		●	

^a^For <15 kgBW, 1 mg/kg to 25 mg/m^2^.

### Response and toxicity evaluation

The primary endpoints were progression-free survival (PFS), overall survival (OS), safety, and peripheral B-cell depletion, whereas response rate was set as a secondary endpoint. At each visit, the dogs were assessed by physical examination, CBC, serum biochemistry, lymph node measurement, and adverse events (AEs) monitoring. Adverse events were recorded based on owner-reported history and clinicopathological evaluations and graded according to the Veterinary Cooperative Oncology Group–Common Terminology Criteria for Adverse Events (VCOG-CTCAE v2).^19^ After the start of 4E1-7-B_f therapy, peripheral B-cell and T-cell counts (CD21+ and CD3+ cells) were monitored in peripheral blood using flow cytometry at every visit, conducted by Animal Allergy Clinical Laboratories Inc. Any dose adjustments or supportive care were administered at the discretion of the attending clinician. Response to treatment was assessed using the VCOG Response Evaluation Criteria for Peripheral Nodal Lymphoma in Dogs,^20^ with the following definitions: complete response (CR), all measurable peripheral lymph nodes returned to a nonpathological size; partial response (PR), at least a 30% reduction in the sum of the widest diameters of peripheral lymph nodes. Stable disease, <30% reduction or <20% increase in peripheral lymph node size and, progressive disease (PD), > 20% increase in peripheral lymph node size, or new lesion appearance.

Upon completing the modified UW25 protocol, the dogs were monitored for recurrence every 4 weeks for 1.5 years and thereafter at variable intervals determined by the attending clinician, typically every 2-3 months depending upon each dog’s condition and the clinical judgment of the attending clinician. Recurrence was assessed by history, physical examination, lymph node measurements, and CBC. Dogs were removed from the study if they received additional treatments that affected tumor size, such as other chemotherapy, immunosuppressive drugs, radiation therapy, or surgery; if they had clinically relevant protocol deviations; if AEs occurred that, in the judgment of the attending veterinarian, warranted discontinuation of study participation (eg, severe systemic illness or treatment-related severe complications); if the owner withdrew consent; or, if the tumor showed clear progression. Upon withdrawal, dogs could receive additional treatment as determined by the attending clinician. After relapse or disease progression, rescue chemotherapy protocols were administered at the discretion of the attending veterinarians. For dogs that had completed the full CHOP + 4E1-7-B_f protocol, CHOP re-induction commonly was attempted after relapse. In contrast, for dogs for which the protocol was discontinued prematurely, alternative rescue regimens were selected based on individual clinical considerations. The variability in post-relapse treatment was not standardized and was influenced by the clinician’s judgment and owner preferences.

### Statistical analysis

Continuous data are presented as median and range, and categorical data are expressed as frequencies and percentages. The primary efficacy endpoints were the objective response rate, PFS, and OS. Objective response rate was defined as the percentage of dogs achieving CR or PR. Progression-free survival (PFS) was defined as the time from treatment initiation to disease progression or death from any cause. Overall survival (OS) was defined as the time from treatment initiation to death from any cause. Dogs still alive at the time of data analysis were censored from the OS analysis. The Kaplan–Meier analysis was performed to estimate and display OS and PFS distributions. All statistical analysis was performed using JMP Pro18.0.2 (JMP Statistical Discovery LLC).

## Results

### Patient population

The study enrolled 13 client-owned dogs with previously untreated multicentric B-cell lymphoma (Table 2). All dogs had a confirmed B-cell immunophenotype, as assessed by CD21 and CD20 expression on the malignant lymphocytes, collected by FNA. All dogs were evaluated for response, OS, PFS, and AEs. Data on age, weight, approximate stage, substage, and immunophenotype are presented in Table 2. The most common breed enrolled was Jack Russell Terrier (*n* = 3), followed by 2 of each of the French Bulldog, Miniature Dachshund, and mixed breed dog. Single dogs of each of the following breeds were also enrolled: Golden Retriever, Toy Poodle, Shetland Sheepdog, and American Cocker Spaniel. There were 7 females (1 intact, 6 spayed) and 6 males (1 intact, 5 neutered). The median age of the dogs was 9 (range, 2-15) years.

**Table 2:** Baseline characteristics of dogs (*n* = 13).

**Age, median (range), years**		9	(2-15)
**Weight, median (range), kg**		6.6	(3.94-31.25)
**Sex, *n* (%)**			
	Male	6	(46.1)
	Female	7	(53.8)
**Stage, *n* (%)**			
	3	2	(15.4)
	4	2	(15.4)
	5	9	(69.2)
**Substage, *n* (%)**			
	a	10	(76.9)
	b	3	(23.1)
**Steroid pretreatment, *n* (%)**			
	No	11	(84.6)
	Yes	2	(15.4)
**Dose reduction, *n* (%)**			
	No	7	(53.8)
	Yes	6	(46.2)
**Dose delay, *n* (%)**			
	No	3	(23.1)
	Yes	10	(76.9)

Of the 13 dogs enrolled, 2 had stage III B-cell lymphoma (*n* = 1 substage a, *n* = 1 substage b) and 2 had stage IV (*n* = 1 substage a, *n* = 1 substage b) at diagnosis. An additional 9 dogs had stage V (*n* = 8 substage a, *n* = 1 substage b). However, not all dogs underwent bone marrow aspiration; many were staged based on the presence of abnormal lymphocytes in the peripheral blood alone.

### 4E1-7-B_f combined with CHOP therapy and overall treatment response

**Figure 1:**
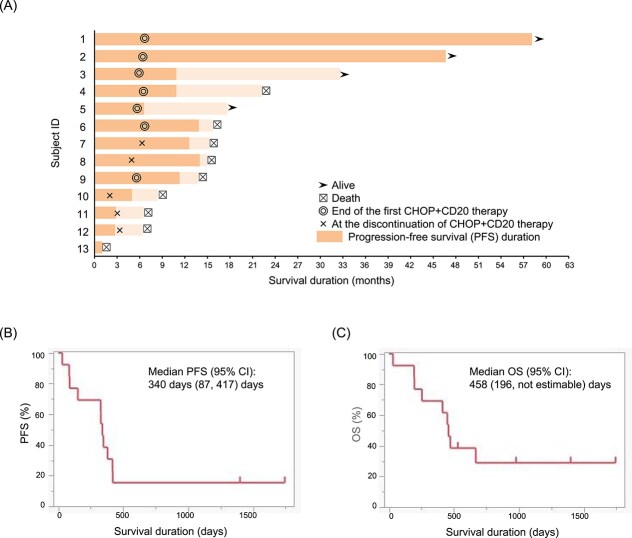
Clinical outcomes of canine lymphoma patients treated with the combination of 4E1-7-B_f and CHOP therapy. (A) Swimmer plot depicting the treatment and survival timeline of 13 dogs with lymphoma treated with 4E1-7-B_f in combination with CHOP chemotherapy. The dark bars and the light bars represent the progression-free survival (PFS) duration and survival after the PFS duration, respectively. Symbols indicate key treatment events:  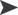 still alive;  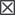 death;  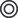 end of first 4E1-7-B_f and CHOP therapy; × dropout of 4E1-7-B_f and CHOP therapy. (B) Kaplan–Meier survival curve for PFS in days. The median PFS was 340 (95% CI, 87-417) days. (C) Kaplan–Meier survival curve for overall survival (OS) in days. The median OS was 458 (95% CI, 196—not estimable) days. Abbreviations: CHOP = cyclophosphamide, doxorubicin, vincristine, and prednisone.

Seven dogs (53.8%) successfully completed the designated 25-week CD20–CHOP protocol (Figure 1A and Table S1). Among the 6 dogs that did not complete the protocol, 2 (cases 11 and 12) developed PD after 3 months of treatment, 3 (cases 7, 8, and 10) the treatment protocol discontinued because of clinically relevant AEs. Dogs 7 and 8 had high kidney function test results and dog 10 developed a hepatic abscess, possibly secondary to febrile neutropenia after cyclophosphamide administration. These AEs were assessed by the attending clinicians as requiring withdrawal from the study. Thus, 3 cases (23.1%) had treatment discontinued because of AEs. One dog (case 13) died at week 4, 3 days after receiving mitoxantrone. The dog presented to an emergency hospital in cardiopulmonary arrest, and the attending veterinarian suspected airway obstruction associated with vomiting as the cause of death. However, aspiration pneumonia was not definitively confirmed, and thus the causal relationship with therapy remains unclear.

In terms of treatment response, all 13 dogs (100%) achieved CR as their best response, with a median time to CR of 3 (range, 2-11) weeks. Two dogs (cases 1 and 2) remained in CR at the last follow-up after completing a single 25-week treatment cycle (Figure 1A). Dogs that experienced PD and were removed from the study were monitored for overall outcomes, including OS, and serial blood samples were collected to assess circulating B- and T-cell populations. Of the 11 dogs that eventually experienced disease progression, 7 received additional rescue chemotherapy, including CHOP re-induction, lomustine (CCNU), nimustine (ACNU), l-asparaginase, or single-agent vincristine or cyclophosphamide. The treatment decisions were based on the judgment of the individual attending veterinarians. Detailed information on rescue regimens and post-progression outcomes is provided in Table S1.

Median PFS for all dogs was 340 (95% CI, 87-417) days (Figure 1B). Of the 13 dogs enrolled, two were censored in the PFS analysis because they had not experienced disease progression at the time of data cutoff. The median OS for all dogs was 458 (95% CI, 196–not estimable) days (Figure 1C), with 4 dogs censored in the OS analysis because they were still alive at the time of analysis. The 1-year and 2-year survival rates were 69.23% and 38.94%, respectively.

### Adverse events

Seventy three doses of 4E1-7-B_f were administered to the dogs analyzed; seven received seven administrations, two received six administrations, two had four administrations, one had three administrations, and one had one administration (Table S1). During the first cycle of the combination of 4E1-7-B_f and CHOP therapy, 84 AEs were documented across 13 dogs (Table 3). All dogs experienced at least one AE, but the majority of AEs were grade 1 (38.1%) or 2 (28.6%). The most common grade 3 AE was hyporexia, followed by neutropenia and diarrhea. Grade 4 AEs included neutropenia and high alanine transaminase activity and blood urea nitrogen (BUN) concentration. These toxicities, including increased alanine aminotransferase activity and BUN concentrations, are not commonly associated with CHOP components and therefore cannot be definitively attributed to standard chemotherapy. Although it remains unclear, the possibility that these AEs were partially related to 4E1-7-B_f, especially because of its sustained B-cell depleting effect, cannot be excluded.

**Table 3:** Frequency of common adverse events by grade during CD20–CHOP therapy (*n* = 13).

	G1	G2	G3	G4	G5
**Gastrointestinal**					
**Hyporexia**	3	2	7		
**Diarrhea**	1	6	3		
**Vomiting**	4	4			
**Hematologic**					
**Anemia**	4	4			
**Neutropenia**	0	2	6	4	
**Thrombocytopenia**	3	1			
**Hepatic**					
**Increased ALT**	6	1	1	3	
**Kidney**					
**Increased BUN**	5	2	1	1	
**Increased Cre**	6	2	2		

Abbreviations: ALT = alanine aminotransferase; BUN = blood urea nitrogen; CHOP = cyclophosphamide, doxorubicin, vincristine, and prednisone; Cre = creatinine; G = grade (per VCOG-CTCAE); n = number of dogs.

### B-cell depletion and recovery

**Figure 2:**
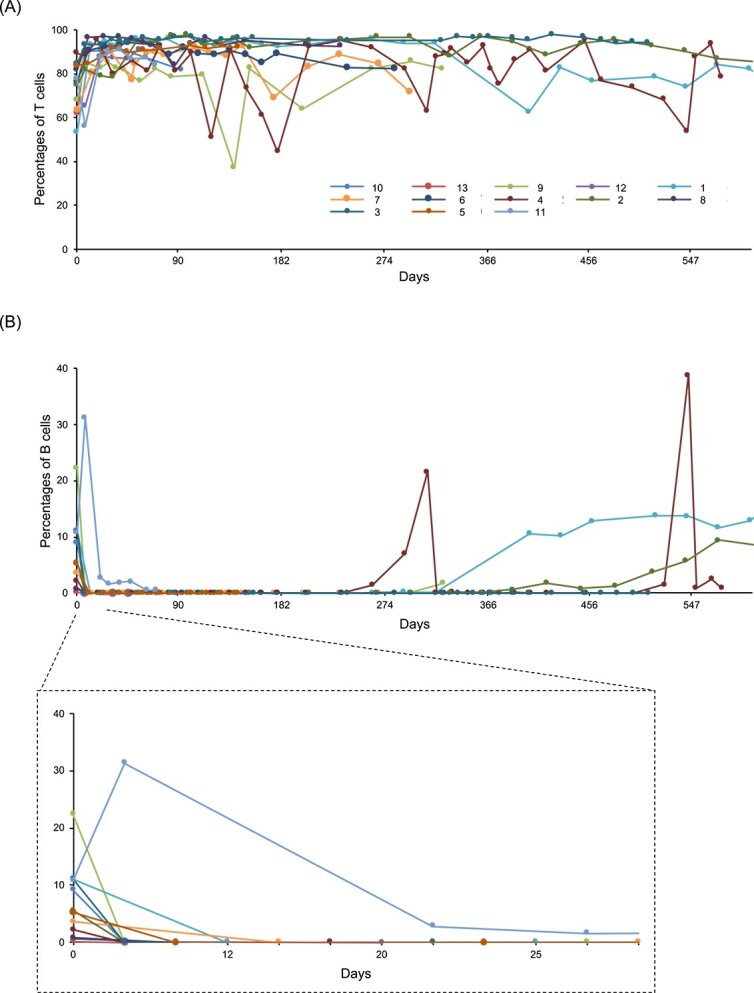
Longitudinal changes in the peripheral blood lymphocyte populations during and after the combination of 4E1-7-B_f and CHOP therapy. (A) Percentage of T-cells in the peripheral blood over time in dogs with lymphoma treated with 4E1-7-B_f in combination with CHOP chemotherapy. (B) Percentage of B-cells in the peripheral blood over time in the same cohort. The inset below shows an enlarged view of the changes from days 0 to 32. Abbreviation: CHOP = cyclophosphamide, doxorubicin, vincristine, and prednisone.

Flow cytometry was used to quantify the percentages of the CD21+ B-cell and CD3+ T-cell populations in the peripheral blood lymphocytes at each visit after week 3 of the protocol (Figure 2). Although the percentages of CD3+ T-cells did not decrease throughout the observation period, despite some fluctuations (Figure 2A), CD21+ B-cells were completely depleted at the first visit after the first anti-CD20 treatment and vincristine (Figure 2B), except for 1 dog (case 11), and B-cell depletion persisted throughout the CD20–CHOP therapy (Figure 2B). The percentages of CD21+ B-cells were monitored in most cases for variable periods after the completion or discontinuation of the combination of 4E1-7-B_f and CHOP therapy. Among them, 3 dogs (cases 11, 12, and 13; Table S1) relapsed and were no longer monitored. However, of the remaining 10 patients, B-cells remained below 1% for > 200 days in all 5 cases monitored for > 300 days, and in 3 of these 5 cases, B-cells remained below 1% for > 300 days, even though no CD20 antibody was administered for > 300 days. The earliest B-cell appearance was 207 days after the end of chemotherapy, and the one case remained below 1% for 333 days, the observation period for B-cell percentage. Of the 5 cases that were monitored for < 300 (median, 85; range, 29-158) days, the B-cell count increased 158 days after the end of chemotherapy. However, B-cells were not detected in all other cases during the observation period. Moreover, the serum concentrations of anti-4E1-7-B_f antibody were measured before, 3 months after, 6 months after, and 1 year after treatment. No anti-4E1-7-B_f antibodies were detected in all but one case (case 6), where no serum was available for measurement (data not shown). On the other hands, notably, all 3 dogs that experienced relapse (cases 3, 4, and 5) still exhibited undetectable CD21+ B-cells in the peripheral blood at the time of disease progression. This observation suggests that peripheral B-cell depletion alone may not reliably reflect remission status or disease recurrence.

## Discussion

Despite small sample size, our results suggest promising outcomes of anti-canine CD20 antibody with CHOP chemotherapy in terms of OS and PFS. Specifically, all 13 dogs achieved CR, with a median OS of 458 days and a median PFS of 340 days. These results appear favorable when compared to those reported in a recent CHOP-only study,^2^ in which the median PFS and OS were 209 and 321 days, respectively. However, their cohort had a markedly higher median body weight (22.7 kg), whereas the median body weight in our study was 6.6 kg, reflecting the typical smaller breed composition of the Japanese dog population. These differences may introduce bias in cross-study comparisons. Notably, a more directly comparable recent study^21^ analyzed a Japanese cohort with a similar body weight distribution (mean approximately 6 kg) and reported median OS of 306 days. Although these data suggest that prolonged survival is achievable in small-breed dogs receiving CHOP-based therapy alone, the consistently high CR rate and sustained B-cell depletion observed in our study raise the possibility that the addition of 4E1-7-B_f may contribute to improved outcomes. Nonetheless, we acknowledge that single-arm design, small sample size, selection bias, treatment setting, and supportive care differences still may confound direct comparisons, and additional investigations using randomized controlled trials are necessary to fully assess the advantages of this combination therapy over CHOP therapy alone.

Blontuvetmab, the first anti-canine CD20 antibody drug, was temporarily marketed by Aratana but no published studies have yet documented its efficacy in dogs with lymphoma. A previous study^16^ was the only published study on anti-canine CD20 antibodies in dogs with lymphoma. That study however combined doxorubicin with investigational immunomodulatory agents such as TAK-981, RV1001, or KPT-0274, the efficacy of which in dogs with lymphoma is not well established. Therefore, the specific contribution of the anti-CD20 antibody remains unclear in that setting. Similar to the previous study,^16^ in which anti-CD20 antibody was administered together with doxorubicin and investigational compounds, our study also did not include a single-agent antibody arm. This feature represents an important limitation, because the specific antitumor effect of 4E1-7-B_f cannot be isolated from the effects of CHOP chemotherapy. However, our study investigated the combination of anti-canine CD20 antibodies with CHOP therapy, and the clinical outcomes were preliminarily assessed using PFS and OS, similar to that of R-CHOP, the standard treatment for DLBCL in humans.

As a single-arm study, our results must be interpreted with caution. Although the CR rate for CHOP monotherapy in dogs with lymphoma typically is reported to be between 65% and 84%,^4^ we observed CR as the best response in all 13 cases. Nevertheless, relapse occurred in 2 dogs ~3 months after treatment initiation, suggesting that not all dogs may benefit from the addition of anti-CD20 antibody treatment. Conversely, the median OS and PFS in our study were 458 and 340 days, both of which are substantially longer than the 301-344 and 238-274 days, respectively, reported in previous studies using CHOP alone.^4^ Moreover, 10 of the 13 dogs in our study were substage a, which may partially explain the better outcomes observed when compared with those in prior reports. In addition, ~85% of the enrolled dogs had not received glucocorticoid treatment before enrollment, which also could have contributed to the favorable outcomes observed in our study. These factors, together with small sample size, may have influenced the observed outcomes. In particular, the small cohort allowed for exceptionally favorable responses in a few individuals to strongly affect the median PFS and OS values, a well-recognized limitation in early-phase veterinary trials.

In our study, 2 dogs achieved long-term remission for > 4 years after just one cycle of 4E1-7-B_f in combination with CHOP, whereas another dog relapsed but maintained long-term remission for nearly 3 years with alternative treatments. The 1-year survival rate for all cases was 69.2%, notably higher than the 36.9%-43.9% reported with CHOP alone in earlier studies.^2^^,^^3^ Whether these improved outcomes are directly attributable to the addition of 4E1-7-B_f remains uncertain, but the distinct mechanism of action of anti-CD20 antibodies, which target tumor cells differently from traditional chemotherapy agents,^17^ could reflect a synergistic benefit from this combination therapy.

Regarding safety, no severe AEs were observed in our study when compared with previous studies of CHOP therapy alone. Infusion-related reactions, which are common in treatments of humans with anti-CD20 antibodies, also were less frequent in our study. A previous study^16^ reported a single infusion-related reaction out of 160 administrations, whereas no infusion-related reactions out of a total of 73 administrations were observed in our study. This difference could be attributed to pretreatment with diphenhydramine and prednisolone or to the nature of the antibody used, which is a type II anti-CD20 antibody.^22^ Type II anti-CD20 antibodies have relatively low CDC but very strong ADCC. One study suggested that infusion-related reactions may be linked to CDC activity,^23^ which could explain the lower frequency of such reactions in our study. On the other hand, it is true that prednisolone use may interfere with ADCC activity. However, because prednisolone was administered only once before each infusion and the antibody used has a half-life of ~3 weeks, we do not believe that this factor would have resulted in sustained inhibition of ADCC. Furthermore, B-cell depletion was consistently observed even in the presence of prednisolone pretreatment, suggesting that any potential suppressive effects on ADCC were minimal and unlikely to have substantially influenced the therapeutic outcome.

Our study also confirmed B-cell depletion, a known AE of anti-CD20 antibody therapy, indicating that the antibody is functioning. Similar to our previous findings, where a single administration of this antibody led to complete B-cell disappearance in beagles within 2-3 weeks,^17^ all but one of the dogs in the our current study exhibited complete disappearance of B-cells after treatment. In dog 11, B-cell counts initially increased, likely because of tumor cells being mixed in, suggesting transient tumor progression. However, the B-cell count steadily decreased thereafter, and in all cases, it remained at zero for the duration of the combined treatment. Even after treatment cessation, B-cell depletion persisted for ~300 days in many dogs, which was unexpected. In contrast, treatment with the 1E4-cIgGB antibody led to only a mild decrease in B-cell count,^16^ indicating that 4E1-7-B_f has notably higher efficacy. Chronic B-cell depletion is similar to the time required for B-cells to recover after rituximab administration in humans.^24^ Interestingly, none of the dogs demonstrated increased susceptibility to infections despite chronic B-cell depletion, suggesting that sustained B-cell depletion does not result in severe immunosuppression in dogs. However, it should be noted that, unlike humans, dogs generally have low rates of chronic viral infections, which may limit the opportunity to observe potential AEs of immunosuppression. In addition, one dog (case 7) developed a hepatic abscess during the treatment period. Although causality could not be established, this finding raises the possibility that chronic B-cell depletion may have contributed to increased susceptibility to opportunistic infections in some dogs and warrants further investigation. The time required for B-cell recovery varied among dogs, and the underlying causes of this variation remain unclear. Resistance mechanisms, such as the loss of CD20 expression on tumor cells,^25^ decreased ADCC activity because of the low activity of macrophages and natural killers cells,^26^ and production of neutralizing antibodies against anti-CD20 antibodies^27^ have been noted in humans treated with anti-CD20 antibodies. In our study, neutralizing antibodies were not detected in all 12 dogs with known anti-4E1-7-B_f antibody concentrations, making it very unlikely as the cause. Other factors were not explored in our study, requiring additional investigations into the reasons for the differences in outcomes between long-term survivors and treatment-resistant cases. However, 2 dogs experienced relapse despite sustained depletion of peripheral B-cells, indicating that peripheral blood monitoring alone may not be a reliable surrogate marker of disease control. Possible explanations include insufficient antibody penetration into lymphoid tissues, emergence of CD20-negative or epitope-modified tumor variants, or local immune evasion in lymph nodes. Nonetheless, the early and profound depletion of peripheral B-cells observed after initial treatment likely reflects on-target biological activity and may serve as a useful surrogate marker for antibody engagement during the initial phase of treatment.

Although a single-agent study of 4E1-7-B_f would more directly clarify its antitumor efficacy, such a trial has not been conducted to date. Given that CHOP remains the standard-of-care treatment for high-grade B-cell lymphoma and is associated with substantial survival benefits, we consider it ethically inappropriate to withhold treatment in favor of experimental monotherapy, particularly in client-owned animals. Therefore, our study focused on assessing the combination of 4E1-7-B_f with CHOP. Although this design limited our ability to isolate the antibody’s efficacy, the results provide encouraging preliminary evidence that warrants further investigation through controlled, prospective trials.

In conclusion, we evaluated PFS and OS in dogs with high-grade B-cell lymphomas treated with anti-canine CD20 antibody combined with CHOP therapy. Although the small sample size and single-arm design prevented definitive conclusions about the superiority of this treatment over CHOP therapy alone, the results are encouraging. The sustained B-cell depletion and observed survival times, which appear comparable to or possibly longer than those reported with CHOP therapy alone in previous studies, suggest that anti-CD20 antibody treatment may have therapeutic potential for dogs with high-grade B-cell lymphomas. However, the small sample size must be emphasized because a few long-term responders may have disproportionately affected survival estimates. Additional investigations using larger, randomized double-blind studies are needed to confirm our findings.

## Supplementary Material

aalaf039_2025-1102_Supplementary_Table_1

## Off-label antimicrobial declaration

Authors declare no off-label use of antimicrobials.

## Institutional animal care and use committee or other approval declaration

Approved by the Japan Small Animal Medical Center Ethical Committee.

## Abbreviations

ADCC: antibody-dependent cellular cytotoxicity
AE: adverse event
BUN: blood urea nitrogen
CD: cluster of differentiation
CDC: complement-dependent cytotoxicity
CHOP: cyclophosphamide, doxorubicin, vincristine, and prednisone
CI: confidence interval
CR: complete response
DLBCL: diffuse large B-cell lymphoma
FDA: Food and Drug Administration
IgG: immunoglobulin G
NaCl: sodium chloride
ORR: objective response rate
OS: overall survival
PD: progressive disease
PFS: progression-free survival
PR: partial response
UW25: Modified University of Wisconsin 25-week protocol
VCOG: Veterinary Cooperative Oncology Group
VCOG-CTCAE: Veterinary Cooperative Oncology Group–Common Terminology Criteria for Adverse Events
